# Authors' Reply: Don't Let the Hypothesis Slip

**DOI:** 10.1371/journal.pmed.0020147

**Published:** 2005-05-31

**Authors:** Ben A Lopman, Geoff P Garnett, Simon Gregson, Simon Gregson, Peter R Mason

**Affiliations:** **1**Imperial College LondonLondonUnited Kingdom; **2**Imperial College LondonLondonUnited Kingdom; **3**Biomedical Research and Training InstituteHarareZimbabwe; **4**Biomedical Research and Training InstituteHarareZimbabwe

In 2003, Brody and colleagues called for researchers to publish analyses investigating the hypothesised importance of medical injections in the transmission of HIV in Africa [1,2]. Considering the general failure of HIV/AIDS control programs and the neglect of this subject, we believed they were right to raise this controversial hypothesis, so we added a question to our field survey and performed a fresh analysis to test the strength of association between injections and HIV incidence [3]. Therefore, we are disappointed that Brody and Potterat think that we are “transparently invested in dismissing” the hypothesis [2].

Now that pertinent incidence data (where the timing of exposure and event can be determined) have been published for Manicaland, Zimbabwe, and Rakai, Uganda, and have shown a lack of association with injections, we think it is unfair to belittle the difficulty of collecting data and to claim that we have not gone to great lengths to collect high-quality data on sexual behaviour [3,4]. On the contrary, because of the general problems in generating reliable responses to questions about sexual behaviour [5,6], the Manicaland HIV/ STD Prevention study has pioneered the use of informal, confidential voting interview methods [6]. Brody and Potterat state that we found “that sexual behaviour was unrelated to risk of incident HIV”. However, in women, having a history of STD symptoms, having multiple sexual partners, or being widowed/separated/divorced (a proxy that a previous sexual partner died of HIV) were associated with HIV incidence. In men, the associations of HIV and sexual behaviour did not reach statistical significance because of the small number of seroconversions.

It is true that women with one reported sex partner did not have a higher incidence than women with no reported partners. However, Brody and Potterat fail to point out that women with multiple sex partners had the highest incidence (31.3 cases per 1,000 person-years) and that rates were lower in men with no sex partners (3.1) than in those with one sex partner (13.6) or multiple sex partners (14.9). These analyses were performed on only a subset of the Manicaland cohort, but other publications have demonstrated the role of sexual behaviours as risk factors for HIV in this population [7].

**Figure 1:**
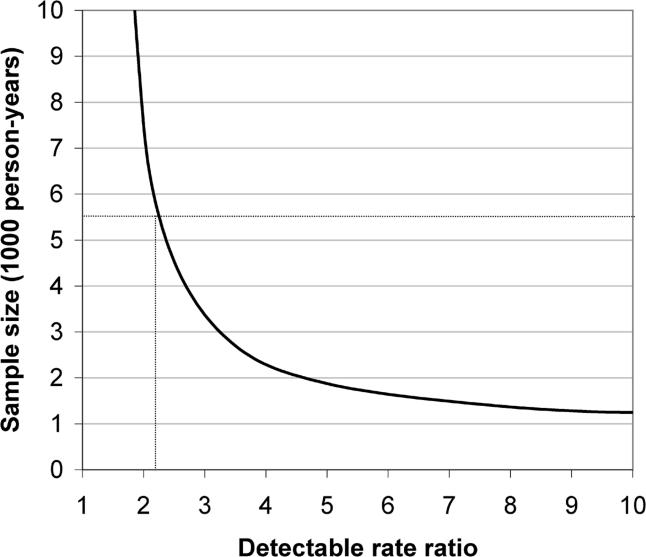
Plot of the Detectable Rate Ratio as a Function of Sample Size Assuming power of 90% at the 95% significance level [11], a rate ratio of 2.27 or greater would have achieved a significant result given the 5,500 years of observation in the sample: 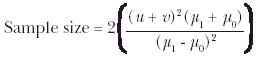 where *u* = 1.28, which is the one-sided percentage point of the normal distribution corresponding to 100% minus the power; *v* = 1.96, which is the percentage point of the normal distribution corresponding to the two-sided significance level; and *μ*1 and *μ*2 are the seroconversion rates in exposed and unexposed individuals, respectively.

We agree that our measure of injections was not perfect, and Brody and Potterat reiterate many of the limitations discussed in our paper: we used a binary (yes/no) measure of exposure, which did not capture the number of injections and had a fairly long follow-up period of three years. These dimensions are being measured in the next round of the cohort study. In the published data, it is possible that some cases had their exposure misclassified, but as many as 40 (60%) of the individuals who seroconverted reported not to have received an injection. Post hoc power calculations (Figure 1) demonstrate that if there was a risk of 2.27 associated with injections, the finding would have been statistically significant. The crude rate ratio for both sexes was 1.1 (95%; confidence interval: 0.7–1.8), which is not evidence that injections are a major transmission route of HIV.

Brody and Potterat also claim that our statistics are flawed because we controlled for age in the analysis. This is a moot point. We presented both univariable and age-adjusted rate ratios of injection exposure—neither showed an association.

We find it strange that Brody and Potterat reference themselves for a study performed in our “own backyard”, which was actually the baseline survey for our current study, and then mislead by saying that it shows little association between sexual behaviour and HIV risk. Lifetime number of sexual partners was in fact a very strong determinant of HIV status in this population [6,7].

In their separate response, Naveed Zafar Janjua and colleagues point out a number of important aspects concerning injection epidemiology and health care–associated infections [8]. First, there is, by definition, a difference between safe and unsafe injections. Second, heightened risks may be associated with “minor and major surgical procedures, dental instrumentation, and tattooing or other traditional practices involving scarification”. (Although not part of our original report, 16 HIV-negative individuals reported to have received a blood transfusion in the follow-up period. None of them seroconverted.) Third, a “needle prick” is a general term that captures lacerations with solid needles as well as those with a borehole. And fourth, the risk associated with receiving one injection is not the same as that for multiple injections, with certain types of injections carrying more risk than others.

However, these concerns expressed by Janjua et al. are not pertinent to the hypothesis that we were testing: are injections a major route of transmission of HIV in this population in Manicaland Province in Zimbabwe? This analysis was motivated by the arguments of Gisselquist et al. that injections are the main driver of HIV transmission in southern Africa [1]. To be clear—we were not testing whether exposure to contaminated needles is a risk factor (clearly it is), whether certain types of injections carry more risk than others (clearly they do), or whether needles are a driver of the epidemic in certain populations in the world (clearly they are). The fact that there is no evidence of association between receipt of injections (of any number) and HIV incidence, before and after controlling for confounding variables, allows us to conclude that injections “do not play a major role in the transmission of HIV in rural Zimbabwe”[3].

The global HIV problem is not a single epidemic. In eastern Europe, over 50% of HIV infections are among users of injection drugs [9]; in Pakistan, people receive on average eight injections per year compared with about one in sub-Saharan Africa [10]. Our findings apply to Manicaland and may be relevant for similar epidemic patterns in southern Africa. They are not generalisable to all locations, but they do refute the hypothesis that HIV is transmitted through medical injections in the study population.
